# Matrix metalloproteinase 9 is associated with conjunctival microbiota culture positivity in Korean patients with chronic Stevens-Johnson syndrome

**DOI:** 10.1186/s12886-022-02406-x

**Published:** 2022-04-19

**Authors:** Jayoon Moon, Yunjin Lee, Chang Ho Yoon, Mee Kum Kim

**Affiliations:** 1 Laboratory of Ocular Regenerative Medicine and Immunology, Seoul Artificial Eye Center, Seoul National University Hospital Biomedical Research Institute, Seoul, Republic of Korea; 2 Department of Ophthalmology, Saevit Eye Hospital, Goyang, Republic of Korea; 3 Department of Ophthalmology, Seoul National University College of Medicine, 103 Daehak-ro, Jongno-gu, Seoul, 03080 Republic of Korea

**Keywords:** Conjunctiva, Matrix metalloproteinase 9, Microbiota, Stevens-Johnson syndrome

## Abstract

**Background:**

Stevens-Johnson syndrome (SJS) is an abnormal immune-response causing extensive exfoliation of the mucocutaneous tissue including conjunctiva. While several factors are associated with the alteration of conjunctival microbiota, the conjunctiva of SJS patients are found to harbor a different microbiota compared to healthy subjects. We investigated the conjunctival microbiota of Korean SJS patients, and identified factors associated with the conjunctival microbiota and its positive culture.

**Methods:**

Medical records were retrospectively reviewed in 30 chronic SJS patients who had undergone conjunctival swab culture sampling. Demographic factors, chronic ocular surface complications score (COCS), tear break-up time (TBUT), tear secretion, tear matrix metalloproteinase 9 (MMP9), and results of conjunctival swab culture were assessed.

**Results:**

Positive culture was seen in 58.1%. Gram positive bacteria was most commonly isolated, among which *Coagulase-negative Staphylococci* (45.5%) and *Corynebacterium species* (40.9%) were predominantly observed. Tear MMP9 positivity was observed significantly more in the positive culture group (100%) compared to the negative culture group (70%) (*P* = 0.041). Topical cyclosporine and corticosteroid were not associated with repetitive positive cultures. No significant differences in COCS, TBUT, and tear secretion were found between culture-positive and culture-negative groups.

**Conclusion:**

Our study suggests that tear MMP9 positivity may be related with the presence of an abnormal ocular surface microbiota in chronic SJS patients.

## Background

Stevens-Johnson syndrome (SJS) is caused by an abnormal immune-response to drugs or other risk factors and is characterized with extensive exfoliation of the mucocutaneous tissue which can be fatal [1, 2]. Acute and chronic ocular manifestations may lead to chronic sequelae of limbal stem cell deficiency, corneal conjunctivalization, persistent epithelial defect, lid margin keratinization, symblepharon, ankyloblepharon etc. [3–5]. Ocular involvement in SJS can be managed with surgical interventions and several medical therapies which includes topical corticosteroids or cyclosporine [4, 6].

An innumerable amount of microbial communities inhabit the human body including the ocular surface, especially the conjunctiva [7]. A normal conjunctiva exhibits diverse microorganisms that commonly consist bacteria such as *Coagulase-negative Staphylococci*, *Staphylococcus group*, *Corynebacterium*, *Propionibacterium* [7–12]. The conjunctival microbiota can be easily altered depending on factors such as use of contact lens, topical or systemic antibiotics, host’s age, or presence of ocular surface diseases etc. [8, 9, 13, 14]. Higher positive culture rate was observed in elder subjects and those with diabetes [15]. Subjects with dry eye or blepharitis were reported to exhibit different conjunctival microbiota compared to healthy subjects [14–17]. In particular, the conjunctiva of SJS patients were found to harbor a significantly different microbiome and have higher culture-positive rate compared to healthy subjects [8, 11, 14, 18].

While the microbiome of several areas of the body have been studied to affect human diseases, the physiological role of ocular surface microbiome is yet unknown. Still, evidence show that an appropriate balance between the ocular surface microbiome and its mucosal immunity helps maintain the homeostasis of commensal bacteria and prevent opportunistic infections [19]. Likewise, a weakened and damaged ocular surface of SJS patients may be more prone to harbor pathobionts and allow opportunistic infections, which can also be aggravated by surgical interventions or medical therapy such as topical immunosuppressants or antibiotics [19–23].

Herein, we investigated the conjunctival microbiota of Korean chronic SJS patients using conventional swab cultures, and identified factors associated with the conjunctival culture results.

## Methods

### Subjects and study design

This study was approved by the Institutional Review Board of Seoul National University Hospital (IRB No. 2102–014-1193, Seoul, Republic of Korea) and was conducted with adherence to Declaration of Helsinki. The informed consent from patients was waived by the IRB because the study was based on the retrospective review of old charts.

This is a retrospective case-series study of chronic SJS patients with more than 6 weeks of disease duration since onset [24] who had undergone conjunctival swab culture sampling between January 1st, 2019 and December 31st, 2020 at Seoul National University Hospital (Seoul, Republic of Korea). From medical chart review, the following data were collected: 1) general medical history and demographic information, 2) clinical characteristics from ocular examinations including chronic ocular surface complications score (COCS, range, 0–15), tear break-up time (TBUT) and tear matrix metalloproteinase 9 (MMP9) elevation, and 3) conjunctival swab culture results. The eye with the highest COCS was chosen for analysis and if the scores were the same in both eyes the right eye was included. Excluded from analysis were patients under 18 years of age, with active infectious keratitis and with insufficient clinical data, such as conjunctival swab culture results and COCS.

COCS was evaluated according to previous studies in modifications based on the grading system by Sotozono et al. [25, 26]. COCS, ranging from 0 to 15, where 15 indicates the most severe ocular complication, was defined as the sum of the following components’ scores: 1) conjunctival hyperemia (no = 0, yes = 1), 2) decreased tear volume (Schirmer strip test ≤1 mm/min = 1), 3) eyelid involvement (trichiasis, distichiasis, or severe meibomian gland dysfunction: 1 for the presence of each component), 4) corneal involvement (superficial punctate keratitis, corneal thinning, corneal opacity: 1 for the presence of each component), 5) limbal deficiency (partial corneal neovascularization = 1, near total corneal neovascularization with persistent corneal epithelial defect = 2, total conjunctivalizatio*n* = 3), and 6) symblepharon formation (1 for each quadrant involved, a total of 4).

TBUT was evaluated under slit lamp biomicroscopy with cobalt blue filter after application of fluorescein strip. TBUT was measured three consecutive times with a stop watch after each blink. The average of the three measurements was used for analysis.

MMP9 elevation to ≥40 ng/ml was tested using InflammaDry test (RPS Diagnostics; Sarasota, FL, USA) according to the manufacturer’s instruction [27, 28]. The InflammaDry device was gently dabbed at multiple locations of the inferior tarsal conjunctiva with releasing the lid after every 2–3 dabs and allowing the patient to blink. After obtaining sufficient tear sample, the device was immediately loaded onto the test cassette and placed directly into the manufacturer’s provided buffer solution. After 10 min, positivity for MMP9 ≥ 40 ng/ml was indicative when 1 blue line and 1 red line were both present in the device’s test result window [27, 28].

History of prior infectious keratitis was defined as those who experienced active infectious keratitis after SJS development which had resolved by the time the current study’s conjunctival culture was performed.

### Conjunctival swab culture and drug sensitivity test

Conjunctival swab culture was performed initially before applying any eyedrops, including topical anesthesia or fluorescein strips. Conjunctival swab sampling from each eye from deep portions of the medial and lateral lower conjunctival fornix was carried out using a sterilized cotton tip for each site [11]. Careful caution was taken to avoid the sterilized cotton tip from being possibly contaminated by factors, such as the eyelid skin or eyelash. After obtaining each swab sample, it was immediately inoculated directly onto either blood agar plate or Sabouraud’s agar plate. When possible, a repetition of conjunctival swab culture was performed in the same manner at an interval of at least 3 months since last test.

Incubation for the growth of bacteria and fungus, and drug susceptibility tests were performed at the department of laboratory medicine at Seoul National University Hospital (Seoul, Republic of Korea). Blood agar plate was used to culture a wide range of bacteria, including fastidious microbes and those that are difficult to grow such as *Streptococcus* and *Staphylococcus*, and to differentiate hemolytic bacteria. Sabouraud’s agar plate was used in cultivating fungus, such as yeasts and molds, and filamentous bacteria. All collected specimens were incubated at 37 °C (for bacteria) or 30 °C (for fungi) and examined daily for microorganism growth for 1 week and weekly for up to 1 month. Laboratory analyses consisted of culture, microorganism identification, and drug sensitivity tests. Drug sensitivity tests were performed by agar diffusion method using the subsequent antibiotics according to clinical and laboratory standards institute (CLSI) guidelines: ampicillin, oxacillin, penicillin G, amoxicillin/clavulanic acid, imipenem, gentamicin, rifampicin, ciprofloxacin, levofloxacin, moxifloxacin, trimethoprim/sulfamethoxazole, teicoplanin, vancomycin, clindamycin, erythromycin, nitrofurantoin, linezolid, quinupristin/dalfopristin, tetracycline, cefoxitin, ceftriaxone, chloramphenicol, piperacillin/tazobactam, cefotaxime, ceftazidime, cefepime, ertapenem, meropenem, amphotericin, fluconazole, voriconazole, flucytosine [29].

### Statistical analysis

Statistical analysis was performed using SPSS software for Windows version 22.0 (SPSS, Inc., Chicago, IL, USA). Shapiro-Wilk test was used to test the normality of the data. Non-parametric Mann–Whitney test was used to compare between groups because the variables were not normally distributed. Fisher’s exact test or Pearson’s Chi square test was used to compare frequencies of categorical variables as appropriate. A probability value of < 0.05 was considered statistically significant. The results are presented as means ± standard deviations (SDs) unless otherwise indicated.

## Results

### Demographic and ocular characteristics in SJS patients

General demographics and clinical ocular characteristics of the enrolled patients are shown in Table 1. A total of 30 eyes from 30 patients with SJS were assessed. The average age was 47.8 ± 16.5 (18–71) years and the disease onset age was 34.6 ± 17.8 (7–66) years. The disease duration was 13.2 ± 12.1 (0.27–52) years. 9 (30.0%) and 21 (70.0%) patients were male and female, respectively. Cold medications were the most common cause for SJS, followed by antibiotics and other medications, such as antiepileptic drugs. Fourteen (46.7%) patients had past experience of infectious keratitis. Nearly 70% of all patients were using topical medications, such as corticosteroids, cyclosporine or antibiotics, at the time when conjunctival swab culture sampling was performed. Schirmer test revealed an average of 6.3 ± 6.3 mm while the TBUT was 3.4 ± 1.1 s. MMP9 was positive in 24 (80.0%) eyes. The average COCS was 8.4 ± 3.3. It was considered as low COCS when the score ranged between 0 and 7, while a score of 8 or higher was defined as high COCS [25, 26]. 10 (33.3%) and 20 (66.7%) eyes were low and high COCS, respectively.

**Table 1:** General demographics and clinical characteristics

Age (years)	47.8 ± 16.5 (18–71)
Onset Age (years)	34.6 ± 17.8 (7–66)
Disease Durations (years)	13.2 ± 12.1 (0.27–52)
Gender (Male: Female)	9 (30.0%): 21 (70.0%)
Cause of SJS (Cold drugs: Antibiotics: Others)	11 (36.7%): 6 (20.0%): 13 (43.3%)
History of Infectious Keratitis	14 (46.7%)
Initial Use of Topical Medications	
Corticosteroid	21 (70.0%)
Cyclosporine	20 (66.7%)
Antibiotics	20 (66.7%)
Schirmer test (millimeters)	6.3 ± 6.3 (0–30)
TBUT (seconds)	3.4 ± 1.1 (1.4–5.6)
Positive MMP9	24 (80.0%)
COCS (score)	8.4 ± 3.3 (0–14)
Low (0–7) (patients)	10 (33.3%)
High (≥ 8) (patients)	20 (66.7%)

*SJS* Stevens-Johnson syndrome, *TBUT* Tear break up time, *MMP9* Matrix metalloproteinase 9, *COCS* Chronic Ocular Surface Complications Score

**Fig. 1:**
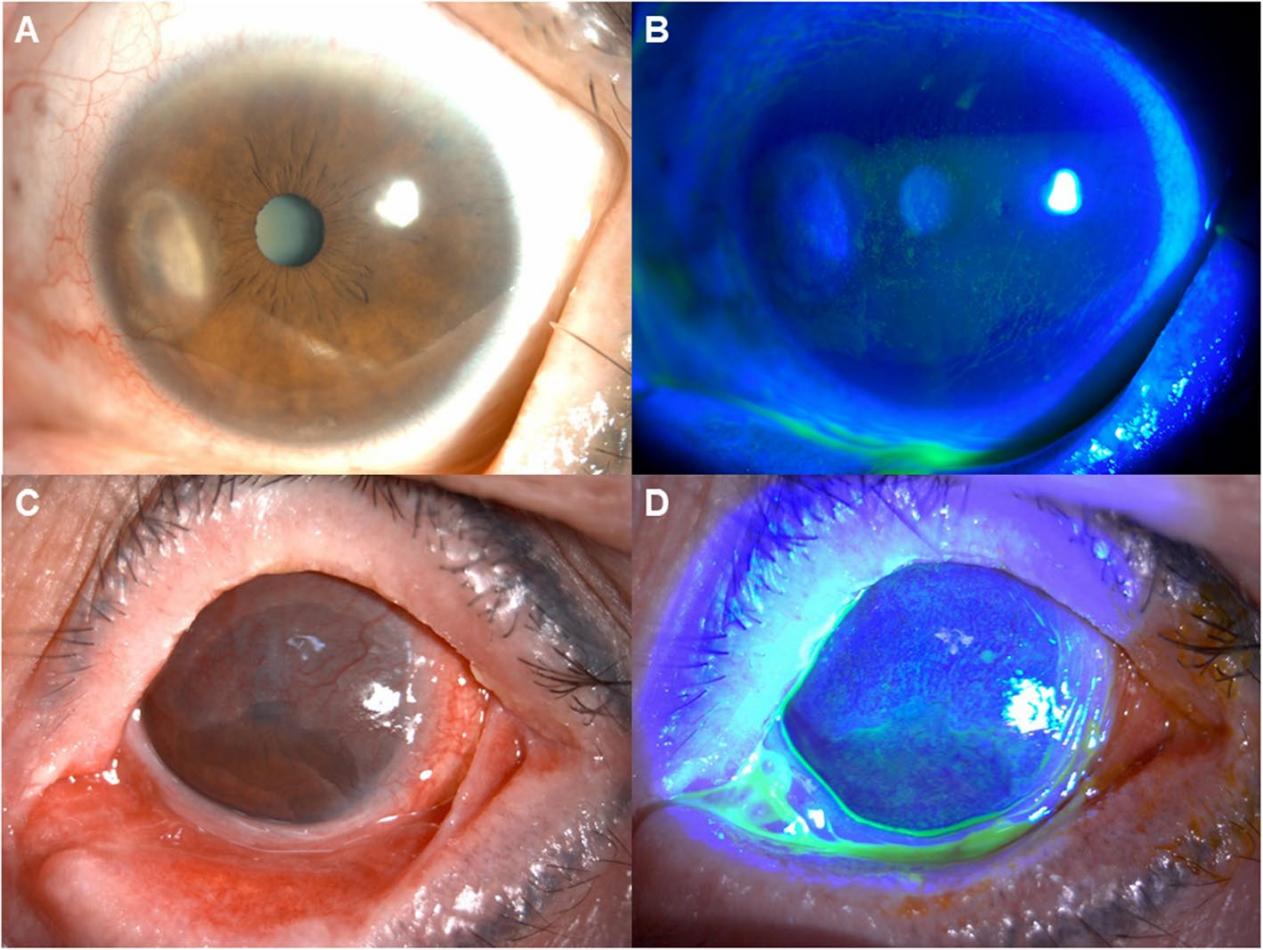
The representative photos of low (0–7) and high (≥ 8) COCS. Figures **A** and **B** represents low (0–7) COCS and are photos of a female subject’s left eye with a COCS score of 3 who had been diagnosed with sulfasalazine-related SJS at the age of 49. Her left eye exhibited 12 clock hours of corneal neovascularization and nasal corneal opacity but without severe meibomian gland dysfunctions, chronic conjunctival hyperemia nor symblepharon (**A**). Under cobalt blue filter examination after fluorescein application, superficial punctate epithelial erosions were observed in the inferior 2/3 of the cornea (**B**). Figures **C** and **D** represents high (≥ 8) COCS and are photos of a female subject’s left eye with a COCS score of 10 who had an onset of SJS at age 44 after taking cold medications. Her left eye had conjunctivalization at the upper 2/3 of the cornea due to limbal stem cell deficiency with diffuse corneal haze, chronic conjunctival hyperemia, severe eyelid meibomian gland dysfunctions with trichiasis and symblepharon at both upper and lower lateral portions (**C**). Under cobalt blue filter examination after fluorescein application, diffuse superficial punctate epithelial erosions were observed (**D**). COCS: Chronic Ocular Surface Complications Score, SJS: Stevens-Johnson syndrome.

The representative photos of low (0–7) and high (≥ 8) COCS are shown in Fig. 1. Figure 1A and B represent low COCS patients and are photos of a female subject’s left eye with a COCS score of 3 who had been diagnosed with sulfasalazine-related SJS at the age of 49. Her left eye was observed to have 12 clock hours of corneal neovascularization, nasal corneal opacity in absence of abnormal eyelids, chronic conjunctival hyperemia and symblepharon (Fig. 1A). Under cobalt blue filter examination after fluorescein application, superficial punctate epithelial erosions were observed in the inferior 2/3 of the cornea (Fig. 1B). Figure 1C and D represent high COCS and are photos of a female subject’s left eye with a COCS score of 10 who had an onset of SJS at age 44 after taking cold medications. Her left eye showed conjunctivalization at the upper 2/3 of the cornea due to partial limbal stem cell deficiency with diffuse corneal haze, chronic conjunctival hyperemia, severe eyelid meibomian gland dysfunctions with trichiasis and symblepharon at both upper and lower lateral portions (Fig. 1C). Under cobalt blue filter examination after fluorescein application, diffuse superficial punctate epithelial erosions were observed (Fig. 1D).

### Isolation and drug resistance results of conjunctival swab cultures

The initial microbial isolation results of conjunctival swab culture samplings are shown in Table 2. 13 (41.9%) patients had no growth of any microorganisms, while 17 (58.1%) patients were observed to have positive culture results. A total of 27 types of different microorganisms were isolated. Among them, 81.5 and 11.1% were gram positive and negative bacteria, respectively, while 7.4% was fungus origin. Among gram positive bacteria, *Coagulase-negative Staphylococci* (45.5%) and *Corynebacterium species* (40.9%) were predominantly observed. Among *Coagulase-negative Staphylococci*, *Staphylococcus epidermidis* (60%) was most commonly isolated followed by *Staphylococcus hominis* (30%). Among gram negative bacteria, *Klebsiella pneumonia* (33.3%), *Stenotrophomonas maltophilia* (33.3%) and *Escherichia coli* (33.3%) were observed. All the isolated fungi were *Candida*.

**Table 2:** Initial microorganism isolation results of conjunctival swab culture

Negative Culture (patients)	13 (41.9%)
Positive Culture (patients)	17 (58.1%)
Total Number of Isolated Microorganisms	27
Gram Positive	22 (81.5%)
*Coagulase-negative Staphylococci*	10
*Staphylococcus epidermidis*	6
*Staphylococcus hominis*	3
*Staphylococcus haemolyticus*	1
*Corynebacterium species*	9
*Staphylococcus aureus*	1
*Streptococcus viridans*	1
*Penicillum species*	1
Gram Negative	3 (11.1%)
*Klebsiella pneumoniae*	1
*Stenotrophomonas maltophilia*	1
*Escherichia coli*	1
Fungus	2 (7.4%)
*Candida*	2

The number of patients according to isolated number of microorganisms are shown in Table 3. Positive culture of a single type of microorganism was seen in 12 (70.6%) of the 17 patients with positive culture results, in which *Corynebacterium species* was most commonly isolated followed by *Staphylococcus epidermidis*. Two and three types of microorganism isolations were observed in 2 (11.8%) and 3 (17.6%) patients, respectively. All fungi were isolated in mixture with other bacteria.

**Table 3:** Number patients according to number of isolated microorganisms

One Isolation (patients)	12 (70.6%)
*Corynebacterium species*	4
*Staphylococcus epidermidis*	3
*Staphylococcus hominis*	1
*Staphylococcus aureus*	1
*Streptococcus viridans*	1
*Penicillum species*	1
*Klebsiella pneumoniae*	1
Two Isolations (patients)	2 (11.8%)
*Corynebacterium species* + *Coagulase-negative Staphylococci*	2
Three Isolations (patients)	3 (17.6%)
*Corynebacterium species* + *Coagulase-negative Staphylococci* *+ Stenotrophomonas maltophilia*	1
*Corynebacterium species* + *Coagulase-negative Staphylococci* *+ Candida*	1
*Corynebacterium species* + *Escherichia coli + Candida*	1

Drug resistance was observed in several microorganisms. Among *Coagulase-negative Staphylococci*, 80% revealed resistance to penicillin, oxacillin or ampicillin, 70% against levofloxacin, moxifloxacin or ciprofloxacin, 50% against erythromycin, 30% against gentamicin, 20% against clindamycin and 10% against tetracycline. Among the isolated *Corynebacterium species*, 66.7% revealed resistance to clindamycin, 55.6% against erythromycin, 33.3% against penicillin, oxacillin or ampicillin, 22.2% against chloramphenicol and 11.1% against gentamicin. However, all isolated *Coagulase-negative Staphylococci* and *Corynebacterium species* were susceptible to vancomycin. The solely isolated *Staphylococcus aureus* was observed to have resistance to ampicillin, penicillin G, gentamicin, ciprofloxacin, levofloxacin, moxifloxacin, clindamycin, and erythromycin, but susceptible to vancomycin and linezolid. The solely isolated *Escherichia coli* was resistant to trimethoprim/sulfamethoxazole and ciprofloxacin, while susceptible to levofloxacin and moxifloxacin. The isolated *Streptococcus viridans*, *Penicillium species*, *Klebsiella pneumonia*, *Stenotrophomonas maltophilia* and *Candida* did not reveal any drug resistance. Changes in drug susceptibility were found in 3 (23.1%) of the 13 patents who had received repetitive conjunctival swab cultures (Table 4). Neither MMP9 positivity nor topical eyedrops (steroid, cyclosporine, or antibiotics) affected change of drug susceptibility among patients with persistent culture positivity (Fisher’s exact test, *P* > 0.05, Table 4).

**Table 4:** Factors associated with changes of drug susceptibility in repeated culture group

	Change of drug susceptibility		*P* value*
	Negative (*n* = 10)	Positive (*n* = 3)	
Initial use of			
Corticosteroid	9 (90%)	2 (66.7%)	0.423
Cyclosporine	7 (70%)	3 (100%)	0.528
Antibiotics	6 (60%)	2 (66.7%)	1.000
MMP9 positivity	9 (90%)	2 (100%)^a^	1.000

*MMP9* Matrix metalloproteinase 9

*Fisher’s exact test

^a^Only two patients had conducted tear MMP9 test

### Tear MMP9 is associated with positive conjunctival swab culture results

**Fig. 2:**
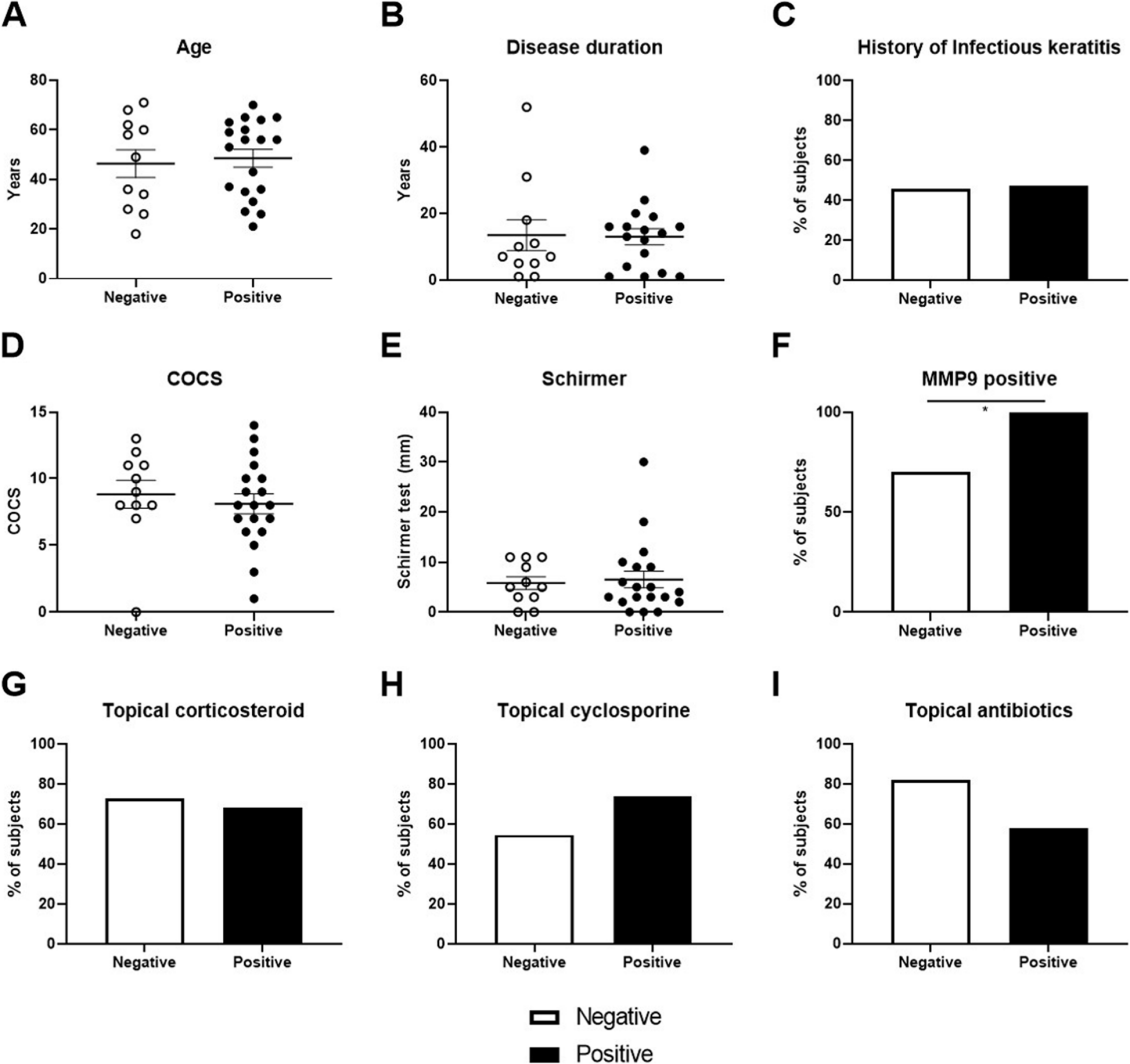
Analysis of demographic characteristics or clinical factors to affect positive culture group. **A**-**C** No significant differences were observed in the average age (**A**), disease duration (**B**) and history of prior infectious keratitis (**C**) between positive-culture and negative-culture groups (Mann-Whitney test, Fisher’s exact test, *P* > 0.05). **D**-**E** The COCS (**D**) and tear secretion by Schirmer test (**E**) were not different between those groups (Mann-Whitney test, *P* > 0.05). **F** Tear MMP9 positivity was higher in positive-culture group than in negative-culture group (Fisher’s exact test, *P* = 0.041) (**G-I**). There were no differences in the use of topical medications, such as corticosteroids (**G**), cyclosporine (**H**) or antibiotics (**I**) (Fisher’s exact test, *P* > 0.05). COCS: Chronic Ocular Surface Complications Score, TBUT: Tear break up time, MMP9: Matrix metalloproteinase 9, vs: Versus

During follow-up, 11 (36.7%) patients had negative culture results (negative group) while 19 (63.3%) patients had positive culture results (positive group). The average ages were 46.4 ± 18.6 and 48.6 ± 15.7 years in negative and positive groups, respectively (Mann-Whitney test, *P* = 0.796, Fig. 2A). SJS onset ages were 32.8 ± 22.2 and 35.7 ± 15.0 years in negative and positive groups, respectively (Mann-Whitney test, *P* = 0.672). The disease durations were 13.5 ± 15.4 and 13.0 ± 9.9 years in negative and positive groups, respectively (Mann-Whitney test, *P* = 0.555, Fig. 2B). The negative group consisted 4 male and 7 female patients, while the positive group included 5 male and 14 female patients (Fisher’s exact test, *P* = 0.687). As a culprit drug, cold medicine was found in 4 patients (36.4%) in positive group and 5 patients (36.8%) in negative group (Fisher’s exact test, *P* = 1.000). The history of prior infectious keratitis was not different between groups (Pearson’s Chi square test, *P* = 0.919, Fig. 2C). There was no difference regarding COCS between groups (negative group 8.8 ± 3.5 versus (vs.) positive group 8.1 ± 3.2, Mann-Whitney test, *P* = 0.352, Fig. 2D). Also, no significant differences in Schirmer test (negative group 5.8 ± 4.2 mm vs. positive group 6.5 ± 7.3 mm, Fig. 2E) and TBUT (negative group 3.5 ± 0.2 s vs. positive group 3.4 ± 1.2 s) were observed between groups (Mann-Whitney test, *P* = 0.696 and 0.866, respectively). During the follow-up period, positive for tear MMP9 was observed significantly more in the positive group compared to the negative group (100.0% vs 75.0%, respectively, Fisher’s exact test, *P* = 0.041, Fig. 2F). There was no difference in the use of topical medications, such as corticosteroids, cyclosporine or antibiotics (Fisher’s exact test, *P* > 0.05, Fig. 2G-I).

A total of 13 patients had repetitive conjunctival swab cultures over an interval of at least 3 months since last sampling. Among them, 3 (23.1%) patients experienced a transition from positive to negative culture results (negative-transition group), while 10 (76.9%) patients had persistent positive culture results (positive-persistence group). The demographics and clinical factors did not differ between groups, including age, history of prior infectious keratitis, COCS, Schirmer test, TBUT, tear MMP9 positivity, and topical medications (corticosteroids, cyclosporine or antibiotics). Disease durations were 5.0 ± 6.9 and 13.9 ± 6.7 years in the negative-transition and positive-persistence groups, respectively, showing a marginal difference though not statistically significant (Mann-Whitney test, *P* = 0.066).

## Discussion

This study presented (1) high conjunctival swab culture positivity rate in Korean chronic SJS patients with predominant isolation of *Coagulase-negative Staphylococci* and *Corynebacterium species*, (2) tear MMP9 positivity to be related with positive culture, and (3) no association between the use of topical cyclosporine or corticosteroid and persistent culture positivity. This study demonstrates high conjunctival swab culture positivity of 58.1% in Korean chronic SJS patients, in which gram positive bacteria prevailed, and *Coagulase-negative Staphylococci* and *Corynebacterium species* were the most commonly isolated microorganisms. Though several microorganisms revealed drug resistance, all isolated bacteria were susceptible to vancomycin and all isolated fungus were susceptible to all antifungal agents. Moreover, neither tear MMP9 positivity nor topical medications, such as corticosteroid, cyclosporine, and antibiotics, was associated with altering drug susceptibility in repetitive culture positive patients.

Several studies observed that the normal conjunctiva harbors diverse microorganisms, which were identified to be mainly composed of *Coagulase-negative Staphylococci*, *Propionibacterium, Corynebacterium species*, *Lactobacillus*, and *Streptococcus*, and can be easily altered by several factors such as age, diabetes, use of contact lens, or presence of ocular surface diseases [7–14]. To date, several methods, such as conventional swab culture or genetic sequencing methods, have aided the identification of these minute amount of microorganisms residing in the conjunctiva [9]. Several studies have reported significant differences in conjunctival microbiota of subjects with ocular surface diseases, especially SJS, compared to those of healthy subjects [8, 11, 14]. With the conventional swab culture method, SJS was found to have positive culture rate of 59–95%, while a healthy conjunctiva exhibits a relatively low positive culture rate of 10–12.9% [8, 9, 11]. Also, Venugopal et al. and Kittipibul et al. observed that SJS patients’ conjunctival swab cultures resulted in more diverse bacterial isolates, including *Corynebacterium species*, *Coagulase-negative Staphyloccoci* and *Streptococcus*, compared to healthy subjects where *Coagulase-negative Staphyloccoci* were mainly isolated [8, 11]. In utilizing next-generation sequencing methods, Kittipibul et al. additionally identified SJS patients’ conjunctiva to harbor a higher species diversity index and a significantly different microbiome composition, such as higher proportions of *Lactobacillus*, *Bacteroides*, *Pseudomonas*, *Staphylococcus*, *Streptococcus*, *Bacillus* and *Acinetobacter*, compared to healthy subjects’ [8]. Also, Zilliox et al., using next-generation sequencing methods, observed that the ocular surface microbiome is very different according to the type of ocular surface disease present and found a distinct conjunctival microbiome composition in SJS patients with predomination of *Staphyloccocus* compared to healthy subjects, but found that the species diversity index was higher in healthy subjects which could have been attributed to the different targeting variable regions used for sequencing [14]. Likewise, this study observed the conjunctival swab cultures of SJS patients to have high positivity of 58.1% with 12 different types of microorganisms isolated. How the ocular surface microbiome and ocular surface immunity interact is yet unknown, but studies have seen the importance of their balance and, therefore, given that SJS patients have generally abnormal ocular surfaces, they are prone to the imbalance that subsequently can induce commensal bacteria to become pathobionts and consequently lead to possible opportunistic infections [10, 19, 20, 22, 23].

Previous studies reported that the conjunctival swab culture positivity of SJS patients was observed in 59–95% [8, 11, 18]. Venugopal et al. found that the most common isolate in SJS conjunctiva was *Coagulase-negative Staphylococci* followed by *Corynebacterium species* and that only 7.6% was observed to have multiple isolates [11]. Likewise, Kittipibul et al. detected gram positive bacteria as the dominant microorganism, where *Corynebacterium species* was the most common bacteria*,* and found that only 10% resulted with multiple isolations [8]. In another study by Frizon et al., they reported similar results of the gram positive bacteria as the main isolated microorganism in SJS conjunctiva where *Coagulase-negative Staphylococci* was the most common bacteria followed by *Corynebacterium species,* but observed multiple isolations in 54% of SJS patients [18]. Similarly to previous studies, this study also observed positive conjunctival culture of SJS patients in 58.1%, where gram positive bacteria was the predominant isolate. Like other studies, *Coagulase-negative Staphylococci* and *Corynebacterium species* were most commonly isolated, and among the isolated *Coagulase-negative Staphylococci*, *Staphylococcus epidermidis* was mainly detected followed by *Staphylococcus hominis* and *Staphylococcus haemolyticus*. However, in this study, the *Streptococcus group* was isolated in 5.9% of SJS patients which was relatively low compared to past studies of 7.7–33.3%. This may be attributed to the relatively older age group included in this study. Cavuoto et al. reported that the *Streptococcus group* proportion is reduced in elder subjects [13]. Also, this study found that the majority of patients had a single type of bacteria isolated while only 29.4% of patients had more than one type of microorganism isolated. This number of isolations did not differ according to other clinical factors, such as COCS, MMP9 or use of topical corticosteroids or antibiotics.

While Venugopal et al. observed that bacteria isolated from healthy conjunctiva revealed drug sensitivity to most antibacterial agents, they identified all isolated bacteria from SJS conjunctiva to be sensitive to gatifloxacin and moxifloxacin, but resistant against ciprofloxacin in *Coagulase-negative Staphylococci,* ciprofloxacin, tetracycline, cefazolin, and moxifloxacin in *Escherichia coli,* and tobramycin and gentamicin in *Streptococcus pneumonia* [11]. Also, they reported that *Streptococcus viridans* had the highest percentage of drug resistance [11]. Frizon et al. identified low sensitivity to neomycin and penicillin in the *Staphylococcus* group, only 33% sensitivity to chloramphenicol in gram negative bacilli and observed the highest antimicrobial resistance in the *Streptococcus* group [18]. However, this study found the highest percentage of drug resistance in *Coagulase-negative Staphylococci,* where more than half were resistant to penicillin, oxacillin, ampicillin, levofloxacin, moxifloxacin or ciprofloxacin, and did not observe any drug resistance in *Streptococcus viridans*. Also, more than half of the isolated *Corynebacterium species* exhibited resistance to clindamycin and erythromycin. Aside from *Escherichia coli* that was resistant to trimethoprim/sulfamethoxazole and ciprofloxacin, gram negative bacteria from this study did not reveal any drug resistance. The difference in the drug susceptibility test results regarding the *Streptococcus group* compared to previous studies may be because of the low culture rate of *Streptococcus* in this study. Also, the difference in drug resistance of the same kind of microorganism among studies may be attributed to the type of topical antibiotics previously or currently used, in which subjects from Frizon et al. [18] mainly used chloramphenicol eye drops while the patients from this study mainly applied topical levofloxacin or moxifloxacin. Although the drug susceptibility results may differ according to studies, it is still consistently noted that the isolated conjunctival microorganisms from SJS patients have relatively higher percentage of drug resistance compared to healthy subjects. Given that most of the persistent positive cultures did not exhibit any change in drug susceptibility, a presence of an inflammatory ocular surface environment may lead to the abundance of more virulent pathogens with drug resistance in chronic SJS.

To the best of our knowledge, this study is the first to identify the association between tear MMP9 positivity with conjunctival swab culture positivity in SJS patients. Despite recent studies concerning conjunctival microbiota, the role of ocular surface microbiota remains unclear. Moreover, most studies have focused on investigating the compositional or alpha/beta-diversity difference of conjunctival microbiota in subjects with various ocular surface diseases compared to healthy subjects rather than possible relative factors. Though a previous study observed higher culture positivity rate in SJS patients with more severe ocular surface scores [8], this study did not find any significance between COCS and conjunctival swab culture results. In this study, tear MMP9 positivity was more significant in patients with positive results compared to those with culture negative results. Despite a prolonged use of topical corticosteroid or cyclosporine, this high positivity rate of MMP9 implies that the inflammatory environment inflicted by SJS may be more inadequately controlled than expected. A study by Yoshikawa et al. observed increase in interleukin (IL)-8 and Granzyme B in SJS patients with more severe ocular surface scores and found their association with conjunctivalization, neovascularization, opacification or keratinization [30]. Also, the increase in MMP9 level plays a critical role in the development of arthritis, inflammatory disorders, cancer, pulmonary diseases, cardiovascular disorders and dry eye syndrome [31]. MMP9 is highly involved in the disruption of corneal barrier and secretion of MMPs are regulated from the level of gene expression for IL-1β, nuclear factor kappa B (NF-kB) and tumor necrosis factor (TNF)-α [31]. Therefore, tear MMP9 positivity may reflect the presence of ongoing ocular surface inflammation.

High concentrations of MMPs such as MMP2 and MMP9 have been found in not only tears of chronic SJS but also from the skin of acute SJS patients, indicating an important role in pathogenesis of both acute and chronic SJS [32, 33]. Likewise, this study shows high positivity of tear MMP9 in chronic SJS patients and suggests that tear MMP9 positivity may be associated with dysbiotic conjunctival microbiota due to persistent ocular surface inflammation, although MMP9 positivity did not alter drug susceptibility of any isolates. Given that high MMP9 stromal expression can be a prognostic marker in chronic ocular SJS undergoing cultivated oral mucosal epithelial transplantation [34], high tear MMP9 may also be considered as a prognostic marker for ocular surface dysbiosis.

There are some limitations to this study. First, it is limited by the relatively small study size. However, given that SJS is a rare disease, a recruitment of 30 patients is not a small number and is similar to previous studies regarding SJS conjunctival microbiota. Also, this study has value in that this is the first to investigate the conjunctival microbiota in Korean SJS patients. Another limitation is that this study observed the conjunctival microbiota using the conventional swab culture method. Though genetic sequencing methods provide more precise and broader information, conventional swab culture still has the advantage of simplicity and practicality. With concerns of possible re-infection under prolonged use of topical anti-inflammatory agents in eyes with high risks, such as prior infectious keratitis history, we tend to perform conjunctival culture more frequently in those with high risks compared to those with quiescent eyes without any infection history. Therefore, due to the retrospective nature of this study, a possibility of selection bias involving a higher incidence (47%) of prior infectious keratitis history is observed in this study compared to previous reports (35%) [35]. Lastly, this study is limited in that there is no control group. However, previous studies have already found a distinct difference in conjunctival microbiota between SJS and healthy subjects. Still, future studies comparing SJS and healthy subjects’ conjunctival microbiota, and further exploring their associative factors will be beneficial in elucidating the role of ocular surface microbiota.

## Conclusion

In conclusion, this study demonstrated a high culture positivity rate in chronic SJS patients’ conjunctiva. *Coagulase-negative Staphylococci* and *Corynebacterium species* were the most commonly isolated microorganisms. Tear MMP9 positivity was associated with positive conjunctival culture. These findings suggest that tear MMP9 positivity in SJS may be associated with dysbiosis of ocular surface microbiota. Further investigations regarding the associative factors of conjunctival microbiota in SJS patients are necessary in understanding the relation between conjunctival microbiota and ocular surface immunity.

## Data Availability

All data generated or analyzed during this study are included in this published article.

## Abbreviations

COCS: Chronic ocular surface complications score
IL: Interleukin
MMP9: Matrix metalloproteinase 9
NF-kB: Nuclear factor kappa B
SD: Standard deviations
SJS: Stevens-Johnson syndrome
TBUT: Tear break up time
TNF-α: Tumor necrosis factor-α
vs.: Versus
